# Undetected hypertension in primary care – a public health iceberg? Lauren Young

**DOI:** 10.1093/eurpub/ckac129.762

**Published:** 2022-10-25

**Authors:** L Young, H Bibi, R Rasheed

**Affiliations:** Medical Education and Research, Rigg Milner and Corringham Health Centre, East Tilbury, UK

## Abstract

**Background:**

Hypertension is largely asymptomatic and contributes to considerable lifetime cardiovascular morbidity and mortality, costing the NHS £2.1 billion annually. The national prevalence of hypertension is 13.7 % and lack of a national screening programme, despite meeting aspects of the Wilson Junger criteria, adds to delays in detection and treatment. Earlier detection could mitigate future cardiovascular risk. We wanted to understand the potential of detection of prehypertension in primary care to see if this fits the Wilson and Junger criteria for a screening programme.

**Methods:**

GP records of adult patients n = 2178 with a known diagnosis of hypertension on the hypertension register from a practice population of 10,000 patients (prevalence is 22%.) were analysed for the prevalence of prehypertension systolic 120-139 mm hg and diastolic bp of 80-89. The average length of prehypertension, the time delay in detection and treatment were assessed, alongside the prevalence of clinical and therapeutic inertia.

**Results:**

A retrospective analysis of a sample size of 1809 patients out of 2178 patients (83.1%) with known hypertension across 3 primary care sites over 20 years was undertaken. Of these 1809 patients, we found that 1095 patients (60.5%) were prehypertensive prior to being diagnosed. The mean time interval between detection of prehypertension to a formal hypertension diagnosis was 10.6 years, with a standard deviation of 7.89 years with no variation with age or sex. However, 588 patients (32.5%) did not have readings within the prehypertensive ranges prior to diagnosis and were opportunistically detected. 51 patients (2.82%) never had readings recorded within the prehypertensive range.

**Conclusions:**

Prehypertension predates hypertension by an average of 10.6 years. Offering annual screening nationally to patients of risk groups e.g., those with a family history, obesity, and alcohol excess, would enable earlier detection, treatment, and considerable cost saving.

**Key messages:**

• Prehypertension predates hypertension; therefore, hypertension meets the Wilson Junger criteria for earlier detection by a screening programme, which is lacking in the UK.

• Offering a national scheme to screen for hypertension to those at a higher risk, can only be considered a benefit to the public and should be implemented.

